# Evaluating process and effectiveness of a low-intensity CBT intervention for women with gynaecological cancer (the EPELIT Trial)

**DOI:** 10.12688/amrcopenres.12971.1

**Published:** 2021-03-29

**Authors:** Nicholas J. Hulbert-Williams, Lee Hulbert-Williams, Ryan James Flynn, Rosina Pendrous, Carey MacDonald-Smith, Anna Mullard, Brooke Swash, Gemma Evans, Annabel Price

**Affiliations:** 1 Centre for Contextual Behavioural Science, School of Psychology, University of Chester, Chester, CH1 4BJ, UK; 2 North Wales Cancer Treatment Centre, Glan Clwyd Hospital, Bodelwyddan, Rhyl, LL18 5UJ, UK; 3 Ysbyty Gwynedd, Bangor, LL57 2PW, UK; 4 School of Psychology, University of Chester, Chester, CH1 4BJ, UK; 5 Department of Psychological Medicine, Addenbrooke’s Hospital, Cambridge, CB2 0QQ, UK

**Keywords:** Gynaecological cancer, unmet needs, distress, anxiety, depression, low-intensity, cognitive behavioural therapy, quality of life

## Abstract

**Background:**

Improving survival from gynaecological cancers is creating an increasing clinical challenge for long-term distress management. Psychologist-led interventions for cancer survivors can be beneficial, but are often costly. The rise of the Psychological Wellbeing Practitioner (PWP) workforce in the UK might offer a cheaper, but equally effective, intervention delivery method that is more sustainable and accessible. We aimed to test the effectiveness of a PWP co-facilitated intervention for reducing depression and anxiety, quality of life and unmet needs.

**Methods:**

We planned this trial using a pragmatic, non-randomised controlled design, recruiting a comparator sample from a second clinical site. The intervention was delivered over six-weekly sessions; data were collected from participants at baseline, weekly during the intervention, and at one-week and three-month follow-up. Logistical challenges meant that we only recruited 8 participants to the intervention group, and 26 participants to the control group.

**Results:**

We did not find significant, between-group differences for depression, quality of life or unmet needs, though some differences at follow-up were found for anxiety ( *p*<.001). Analysis of potential intervention mediator processes indicated the potential importance of self-management self-efficacy. Low uptake into the psychological intervention raises questions about (a) patient-driven needs for group-based support, and (b) the sustainability of this intervention programme.

**Conclusions:**

This study failed to recruit to target; the under-powered analysis likely explains the lack of significant effects reported, though some trends in the data are of interest. Retention in the intervention group, and low attrition in the control group indicate acceptability of the intervention content and trial design; however a small baseline population rendered this trial infeasible in its current design. Further work is required to answer our research questions, but also, importantly, to address low uptake for psychological interventions in this group of cancer survivors.

**Trial registration:**

ClinicalTrials.gov, NCT03553784 (registered 14 June 2018).

## Plain language summary

An increasing number of people are surviving for longer time periods following treatment for gynaecological cancer and this means we need to change how we care for and support a growing cancer survivor population. Psychological distress and poor quality of life are common in people affected by cancer, and these do not always improve once treatment ends. Providing psychological support can be expensive, which means that not everyone who wants it can access it. Psychological Wellbeing Practitioners (PWPs) have been introduced in UK health care. This workforce might offer an alternative for providing psychological support to a greater number of cancer survivors.

We aimed to test how good a PWP co-delivered intervention is at improving depression, anxiety and quality of life in people who had been treated for gynaecological cancer. The intervention was delivered to small groups of patients over six weekly sessions. We compared those who received the intervention with a similar patient group who did not have access to the same psychological support from a different hospital. Participants reported their psychological wellbeing and quality of life at the point of recruitment, weekly for six weeks, and then at follow-up time-points one week and three months later.

Because of low interest in the group intervention we did not recruit to target. Only 8 participants took part in the intervention, and we recruited only 26 participants in the control group. This means we can’t have full confidence in our results. Nonetheless, the findings indicate that this intervention was helpful for improving participants’ anxiety levels.

Further trials, which recruit a larger number of cancer survivors, are needed to answer our research questions. However, this trial indicates acceptability and potential benefit. We also need to undertake research to understand why so few cancer survivors wanted to take part in this group-based intervention.

## Introduction

Gynaecological cancers (cervical, ovarian, uterine, vaginal and vulvar) accounted for 5.9% (18,026) of all England-based cancer registrations in 2017 ^1^. Many gynaecological cancers are associated with improving survival rates ^2^; however, ovarian cancer, has one of the smallest increases in one- and five-year survival rates compared to other cancer types ^3^. Delay to diagnosis from first symptom presentation is often longer in cervical cancer than other more common cancers ^4^ which may be contributory to comparatively smaller increased in overall survival ^5^. Greater diagnostic delay can also lead to psychological distress ^6^, an important outcome which is understood to be predictive of survival in some cancer groups ^7^. Information on the unmet psychological and supportive care needs of women with gynaecological cancer is limited, particularly comparisons between sub-types of gynaecological diagnosis, and especially in relation to concurrent psychological distress ^8, 9^. Recent research suggests increased fear of recurrence ^10^ and sexuality needs ^11^ may be particularly problematic for this group. Beesley *et al.*’s systematic review 12 suggested that psycho-education may be helpful in supporting gynaecological cancer survivors to manage some late treatment effects; however, more data is needed on the beneficial impacts of psychological treatment on quality of life in people affected by gynaecological cancer ^13^.

Increasing evidence suggests that psychosocial interventions are beneficial in reducing distress in adults with cancer ^14^. Cognitive Behavioural Therapy (CBT) currently enjoys the strongest evidence-base for psychological distress in people affected by cancer ^15^ including interventions which aim to improve anxiety, depression and quality of life ^16, 17^. Concerns remain regarding the generalisability of findings due to small sample sizes used; Xiao *et al.* ^17^ and Hulbert-Williams *et al.* ^15^, for example, emphasise the need for further well-designed and better powered randomised controlled trials to improve the evidence base for psychological interventions in those affected by cancer.

In gynaecological cancers specifically, the evidence is somewhat less conclusive. Beesley *et al*.’s systematic review, concluded that counselling-based approaches improve psychological wellbeing for women with gynaecological cancer ^18^. Further, Rost *et al*.’s comparison of Acceptance and Commitment Therapy (ACT) and CBT for women with advanced ovarian cancer reported improved mood and quality of life in both groups ^19^. Other studies have shown that interventions to increase physical activity can reduce fatigue ^20^ and when combined with CBT, improve quality of life in addition to physical health ^21^. Mindfulness-based interventions have shown promising evidence for improved sexual functioning in those with ovarian cancer ^22^. Null findings for the evidence of psychological intervention in this group have also been reported, however. For example, Chan *et al*.’s randomised controlled trial of CBT-based psychoeducation, stress management, relaxation, and pain and distress management reported no significant effect on quality of life or distress compared with treatment as usual control group receiving routine medical care ^23^. Chan *et al*. postulate that the lack of significant effects may have resulted from the decision not to screen participants for high distress which may have diluted the effect of the intervention, and other authors have noted this as a broader methodological problem in psychosocial oncology intervention science ^15^. 

To maximise potential for implementation into standard care, there is a need for trials of psychological interventions that demonstrate not only evidence for improved outcomes, but also cost- and service-effectiveness too ^24^. Traditional psychological interventions are typically delivered by a small number of trained therapists ^25^ and tend to be time-intensive ^26^. The cost of training, the shortfall of qualified therapists, and the increasing demand for psychological interventions that comes with improved cancer survival rates are making the current service delivery model potentially untenable for long-term sustainability. As such, there a need for the development of more cost-effective delivery models, including those which can be delivered by non-psychologist members of the existing healthcare team.

For some years, the English National Health Service (NHS) has provided the delivery of brief psychological services by a specially trained workforce of psychological wellbeing practitioners (PWPs) as part a stepped-care model within the Improving Access to Psychological Therapies (IAPT) initiative ^27^. Although some questions remain about the overall effectiveness of PWPs ^28^, this model is attractive because of cost-effectiveness potential: by providing lower-intensity, briefer interventions before symptoms escalate in severity, PWPs can have higher caseloads allowing increased access treatment ^29^. Training is also less exhaustive than for other psychological specialists offering potential cost-savings there too. The 2016 NHS Five Year Forward View for Mental Health ^30^, suggests that two-thirds of the increase in target access figures for IAPT will be in individuals with co-morbid physical and mental health conditions. This has acted as a catalyst to expand the PWP role to include service provision for people with chronic health conditions, including cancer. 

An additional cost-benefit element of IAPT services is achieved because of the option for a group-based delivery model ^29^, which is also commonplace in cancer care, including in the delivery of CBT-based psychological interventions ^31, 32^. A recent systematic review of psychotherapeutic interventions for women with metastatic breast cancer concluded that whilst group-based interventions have the strongest evidence or efficacy, they also suffer from some of the lowest uptake and adherence ^33^. Outside of cancer care, research has compared individual versus group delivery of CBT in chronic health settings, finding that group-based delivery leads to greater treatment improvement *and* satisfaction ^34^. Qualitative evaluation of a psychoeducation and counselling group intervention for post-treatment gynaecological cancer survivors suggests that positive impacts were related to the “… special community of mutual understanding and belonging” that is created ^35^.

### Study aims

Stepped-care models such as that used in the IAPT service are recognised as essential parts of good clinical psychosocial cancer care ^36^, but given the changing context in UK-based mental health care, research which explores the efficacy of PWP-supported service delivery on cancer outcomes is needed. Our study reports on a trial of a group-based, CBT intervention for gynaecological cancer survivors that was designed around incorporation of PWPs as co-facilitators. Our primary aim was to explore effectiveness in improving depression and anxiety against a non-randomised control group. As secondary aims, we wanted to test: (a) intervention effects on quality of life and unmet needs, and (b) potential mediators of intervention outcomes.

Although originally planned as a full clinical trial, recruitment difficulties at both our intervention and control site meant that we had to stop the trial short of our recruitment targets. Our paper reports both our originally intended design and adjustments made in relation to our recruitment difficulties. We include discussion of learning points related to this which should be considered in planning similar trails elsewhere.

## Methods

This trial has been registered at ClinicalTrials.gov ( NCT03553784; registered 14 June 2018). The full trial protocol is available on the Open Science Framework (DOI 10.17605/OSF.IO/YGHNE ^37^).

### Design and setting

This study employed a non-randomised controlled trial design. The intervention group were recruited from a gynaecology-specific psycho-oncology service in England, where the intervention is delivered as part of standard care. A pragmatic non-randomised treatment-as-usual control group were recruited from a different clinical setting (North Wales). A fully randomized design was not possible because (a) there was yet no evidence base to roll-out the intervention for delivery at this second recruitment site, and (b) because a single-site randomized trial would have necessitated withholding a standard-care intervention from patients randomized to the control group, which would have raised considerable ethical and logistical concerns ^38^. Pragmatic trial designs ^39^, where data collection has to complement current clinical delivery, are increasingly being used.

The intervention clinical team in the East of England were members of a pilot psycho-oncology service funded by a combination of third sector and local NHS awards. The service was designed to deliver an integrated psychological care model to patients with gynaecological cancers attending a large regional teaching hospital for treatment. The hospital provides comprehensive cancer care to patients both in the county in which it is located, and to out of county patients as a tertiary referral centre. The service was funded to see patients both in outpatient and inpatient settings and was located on the hospital site within the hospital’s department of psychological medicine.

This study was reviewed by the University of Chester Department of Psychology Ethics Committee and was formally approved by the North Wales Research Ethics Committee (REC-4) (Reference: 18/WA/0079; IRAS Project ID: 239518). Health Research Authority permissions were approved at each clinical site. Written informed consent was obtained from all participants to take part in the trial.

### The intervention

The intervention is a group-delivered, low-intensity CBT-based course that is offered as part of standard care to people diagnosed with gynaecological cancer across hospitals in the East Anglia region of England. The content broadly includes: (i) psychoeducation about the emotional impact of cancer survivorship; (ii) values-based behavioural activation; (iii) thought challenging negative and problematic cognitions; (iv) coping with uncertainty; (v) narrative therapy-based approaches to supporting identity concerns; and (vi) psycho-education about sleep hygiene and fatigue. The content of the intervention was not altered by the study protocol as our aim was to evaluate this clinical service as it was designed; some iterative changes were made over time, but these did not change the fundamental therapeutic objectives of the course. The intervention group is co-facilitated by a PWP and a clinical nurse specialist from the medical oncology team. The course delivery is supervised by a clinical psychologist. On design of the service (and research study) the intervention was intended to be delivered over six weekly sessions, however, from group two onwards, the content was spread out to include an additional week with an eighth week added as a wrap-up and review with no new content.

For this trial, the intervention was compared to a treatment-as-usual control group. Participants in this group received no additional intervention other than their standard clinical care and a weekly telephone call with the research for data collection purposes.

### Participants

***Eligibility criteria and sample size.*** Participants were women over 16 years of age who had completed first-line treatment following a gynaecological cancer diagnosis. We excluded those expected not to survive for the full five-month duration of the study (to mitigate against distress), those not fluent in English, and those unable to give informed consent.

Our sample size calculation was based on our original study aims of comparing between-group intervention effectiveness. To adequately power a two-arm experimental comparison (1- *β* = .90; *α* = .95; nine time-points) would have required ten participants per group to detect a medium interaction effect size *f* = .25 (medium effect size are indicated by NICE ^40^ as being necessary for clinical significance in the case of depression-related outcomes), though given that medium effect sizes are uncommon in psychosocial oncology intervention research we planned to recruit in excess of this.

***Recruitment.*** At our intervention-delivery site, patients either self-referred or were informed about the availability of the intervention following completion of first line-treatment by their clinicians using an information sheet. There was no distress screening: all patients accessing the service were offered the chance to join the intervention if they wanted to. Those identified as highly distressed were, however, offered additional one-to-one psychological support as part of stepped-care service provision ^41^. On setting up the service, the expectation was that four or five intervention groups would run each year, each with up to 15 participants per group. We expected that rates of consent for data to be used in our study would be relatively high, and therefore that we would be able to recruit 35–40 participants in a twelve-month period.

At the control sites (two general hospitals in North Wales), eligible participants were identified by clinical teams as part of routine weekly multidisciplinary team meetings. They were then invited to participate by their oncologist or specialist nurse. For this group we included patients prospectively diagnosed through the recruitment period, but also any retrospectively identified who had completed first-line treatment within a period of four months from the start of the study: this was comparable with the intervention group given that the intervention was planned to be delivered just four times per year, thus introducing delay between completing treatment and accessing the intervention. A recent review suggested that with clinician approach we could expect around 70–80% participation rates ^42^, though this is likely to reduce over time due to participant attrition. Our intention was to recruit 100 participants to this group (a) to provide a more representative description of unmet needs in this group, and (b) to enable extraction of a sub-set of participants with similar distress profiles at baseline to the intervention group for matched analysis. For various logistic reasons we were restricted to recruiting our control sample for just a six-month period.

### Data collection and measures

All participants completed self-reported outcome assessments at baseline (pre-intervention), and then weekly for seven subsequent weeks: this was intended to map onto data collection at each week of the intervention delivery, plus a one-week follow-up. From the second delivery iteration of the CBT group, however, an extra two weeks were added to the CBT programme. Given that (a) the majority of our control group data collection was already collected by this point, and (b) the new ‘week eight session’ was a review and wrap-up session only, we did not consider it hugely problematic that this was delivered after our final data collection point: our post-intervention data collection point thus remained at Week 7, which in essence became the final week of delivery in the review programme. The three-month follow-up data was collected via telephone interview conducted by the intervention delivery team. All data for control-arm participants was collected via telephone-based structured interviews at identical time-points to the intervention arm. Patients reported on their own socio-demographic profile. Basic diagnosis and treatment information was provided by the clinical teams, with participant consent.

***Primary outcomes.*** *Depression*. The Patient Health Questionnaire (PHQ-9) ^43^ is a nine-item tool which uses a four-point rating scale (ranging from 0 to 3) to ask how often in the last two weeks participants have experienced symptoms pertaining to appetite, concentration, energy hopelessness, and suicidality. Higher scores indicate higher levels of depression. The PHQ-9 has been established as a valid and reliable measure of depression severity ^43^, and recent work has established it as a sensitive assessment tool for cancer-related distress ^44^.

*Anxiety*. The Generalized Anxiety Disorder questionnaire (GAD-7) ^45^ is a seven-item screening tool to how often in the last two weeks participants have experienced symptoms pertaining to feeling anxious, worried, had difficulty relaxing, and irritability. The GAD-7 uses a four-point rating scale (ranging from 0 to 3) and higher scores indicate higher anxiety. The GAD-7 has been established as an accurate measure of 46, including specifically within psychosocial oncology research ^47^.

Both the GAD-7 and the PHQ-9 were included as they form part of the minimum data set required for the evaluation of IAPT services in England.

***Secondary outcomes.*** *Quality of Life.* The Functional Assessment of Cancer Therapy-General (FACT-G) is a 33-item tool commonly used to assess quality-of-life in patients receiving cancer treatment ^48^. The FACT-G uses a five-point rating scale (ranging from 0 to 4) asking how often in the last seven days difficulties have been experienced across four dimensions of wellbeing: physical; social/family; emotional; functional. Higher scores indicate lower quality-of-life. The FACT-G is a well validated tool for measuring quality-of-life in cancer sample ^49^.

*Unmet needs.* The Short-Form Supportive Care Needs Survey (SCNS SF-34) ^50^ is a 34-item survey to assess level of unmet needs across five separate domains: psychological needs; sexuality needs; health system and information needs; physical daily needs; and, care and support needs. The SCNS SF-34 uses a five-point rating scale (ranging from 1 to 5) asking how often in the last month patients have required help across each of these unmet needs items.

***Therapeutic process measures.*** To evaluate potential intervention mediators, we assessed variables that we expected might be changed by a psychological treatment protocol. To reduce participant burden these were assessed at baseline, post-intervention and follow-up only.

*Thought intrusion.* The Impact of Events Scale-Revised (IES-R) ^51^ is a 22-item measure of thought intrusion following a traumatic event. The IES-R uses a five-point rating scale (ranging from 0 to 4) asking how often in the past week patients have experienced stress reactions after traumatic life events. This is a valid and reliable measure of stress responses in different populations ^52^. We used the thought intrusion sub-scale of the IES-R to measure whether the intervention has reduced the occurrence of problematic cognitions, as would be expected in CBT ^53^.

*Self-efficacy.* Psycho-educational component of the intervention were expected to increase participants’ perceptions of their ability to self-manage the long-term consequences of their cancer and treatment. We assessed this using an 11-item cancer specific self-management self-efficacy scale ^54, 55^ which is an adaptation of the Self-Efficacy for Managing Chronic Disease Scale ^56^, a widely used and well-validated measure. Each item requests a response using a 10-point scale (ranging from 1, not at all confident, to 10, totally confident). The adapted scale for cancer survivors has good reliability and psychometric properties ^54^.

*Psychological flexibility.* Given that the intervention content drew upon themes from third-wave interventions in addition to traditional CBT, we assessed sub-components of psychological flexibility (openness to experience, behavioural awareness, valued action) using the Comprehensive Assessment of Acceptance and Commitment Therapy processes (CompACT) ^57^. The CompACT is a 23-item measure which demonstrates good internal consistency across all three scoring clusters ^57^; high scores indicate greater psychological *in*flexibility.

### Statistical analyses

Analysis was undertaken using SPSS v24. All data were cleaned, recoded and reverse-scored as needed using standard scoring procedures. Where there was less than 10% missing data on self-report variables, mean-score imputation was used. Standard checks for data normality (box plot visual graphs and Shapiro Wilk test of sampling distributions), and sample size determinations, were used to determine whether parametric or non-parametric tests should be used.

Information on recruitment, response and attrition are reported descriptively so as to provide a narrative on the success (or lack thereof) of the study design. Baseline differences in these variables between the intervention and control group were explored using Mann Whitney U tests.

Our sample size was small, and our data were underpowered, but we nonetheless explored some preliminary analyses of our stated study aims. Our primary aim — intervention effectiveness on depression and anxiety — was addressed using 2 by 2 mixed ANOVAs (exploring changes from baseline to post-intervention between condition) and 2 by 3 mixed ANOVAs (exploring changes from baseline to post-intervention *and* follow-up between conditions). Secondary outcomes were also analysed using ANOVAs. Mediation analyses of therapeutic process variables on intervention outcomes was not possible due to limited sample sizes. We therefore used ANOVAs to explore differences between conditions and over-time in these variables instead.

## Results

**Figure 1.**
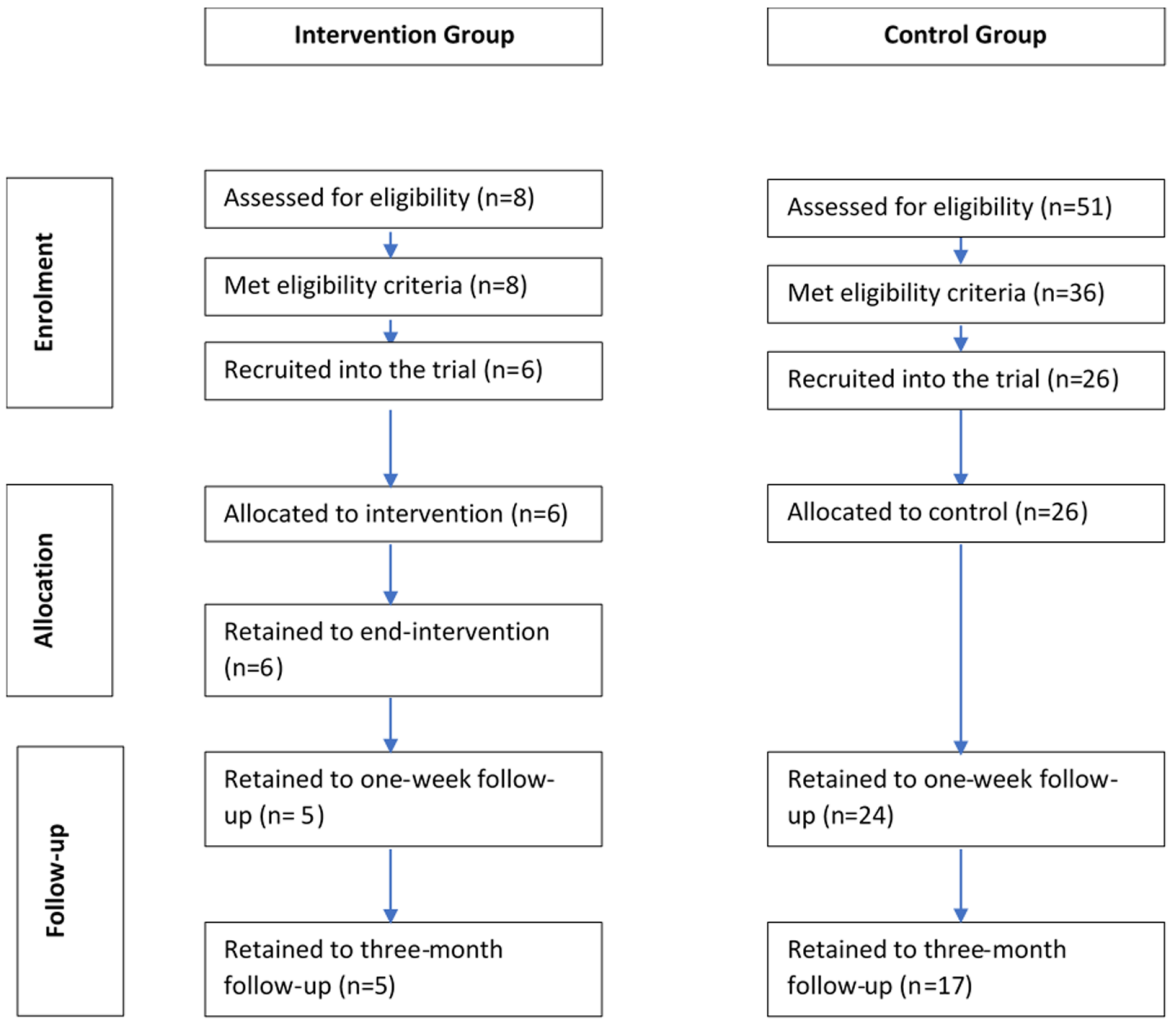
Flow chart of intervention and control group at each stage of the study.

After extending our recruitment period to last almost two years (Recruitment: May 2018 to February 2020; Follow-up period closed in May 2020), we closed recruitment having fallen considerably short of our recruitment targets (see Figure 1). Sign-up for the intervention group was far lower than had been expected, and so fewer intervention groups were run. In total, just eight patients had taken part in one of three iterations of the CBT group. Of these eight, six consented for their data to be used in the study representing a 75% response rate. At our control site, just 26 participants consented to take part; this was a result of both (a) staff sickness at one site leading a period of non-recruitment, and (b) actual diagnosis rates being far lower than had originally been estimated by the local clinical teams. Over our recruitment period, 51 new patients were diagnosed; 36 of these met eligibility criteria (71% eligibility rate), and of those 26 consented for the study (72% consent rate). Although this fell short of our recruitment target (for both groups) it was not possible to keep recruitment open indefinitely; funding to support data collection in North Wales had run out, and due to service re-organisation the group-based intervention in East Anglia was no longer going to be offered as standard care. We thus made the difficult decision to close the study short of target and disseminate our lessons learned (which this paper aims to do). In this context, we decided to conduct a more basic comparison of groups rather than adopting a matched-sample comparison as had been originally planned.

In the CBT group, only one participant completed all sessions and the follow-up. Two participants had sporadic completion of the intervention across all weeks; another participant did not attend Week 5; another missed Week 2 and did not complete follow-up; and two participants missed Weeks 3 and 4, but then resumed participation thereafter. One participant dropped out entirely from Week 4 onwards (overall attrition to follow-up = 16%; see Figure 1).

In the control group, six participants did not complete Week 2; four did not complete Week 3; three did not complete Week 4; three did not complete Week 5; six did not complete Week 6; and eight did not complete follow-up. No participant who completed baseline officially dropped out of the study (overall attrition to follow-up = 35%).

### Sample description

Table 1 summarises key demographic and clinical characteristics of participants. There are some notable differences between the two groups; specifically, the control group were older and therefore more likely to be retired. The majority of our sample (both groups) were white and identified as heterosexual. The control group reported having received less support from local psychology or Macmillan services but this is perhaps because intervention group participants were informed about the study by the dedicated Macmillan-funded service that they were already engaged with. The sample was dominated by participants with ovarian cancer but this did not differ between groups and is representative of the broader gynaecological cancer population. The majority of participants had been diagnosed with Stage 3 cancer, though there was variation in diagnostic staging in both groups. All participants in the CBT group had received treatment with curative intent, whereas some participants in the control group had been treated with palliative intent. The majority of participants had been treated with either surgery alone, or surgery plus chemotherapy.

**Table 1.** Description of demographic and clinical profile of participants.

	CBT ( *n* = 6)	Control ( *n* = 26)
**Age** *M* ( *SD*)	44.50 (13.07)	68.81 (9.52)
**Dependents** *M* ( *SD*)	.17 (.41)	.31 (.55)
**Employment status** Unemployed Employed part-time Employed full-time Self-employed Retired Other	1 (16.7%) 4 (66.7%) 0 0 1 (16.7%) 0	2 (7.7%) 2 (7.7%) 1 (3.8%) 2 (7.7%) 18 (69.2%) 1 (3.8%)
**Relationship status** Yes, living together Yes, living apart No	5 (83.3%) 0 1 (16.7%)	19 (73.1%) 3 (11.5%) 4 (15.4%)
**Ethnicity** White Other	5 (83.3%) 1 (16.7%)	100% 0
**Sexuality** Heterosexual Missing	6 (100%) 0	25 (96.2%) 1 (3.8%)
**Support received** Macmillan information Psychologist None Other	4 (66.7%) 1 (16.7%) 0 1 (16.7%)	11 (42.3%) 0 8 (30.8%) 7 (26.9%)
**Cancer type** Ovarian Peritoneal Endometrial Cervix Ovarian-Endometrial Vulval Missing	4 (66.7%) 0 1 (16.7%) 1 (16.7%) 0 0 0	13 (50.0%) 1 (3.8%) 6 (23.1%) 1 (3.8%) 1 (3.8%) 1 (3.8%) 1 (3.8%)
**Cancer stage at diagnosis** 1a 1b 1c 2a 2b 3a 3c 4a 4b Missing	0 0 1 (16.7%) 0 1 (16.7%) 1 (16.7%) 1 (16.7%) 0 1 (16.7%) 0	5 (19.2%) 3 (11.5%) 1 (3.8%) 1 (3.8%) 1 (3.8%) 2 (7.7%) 7 (26.9%) 2 (7.7%) 3 (11.5%) 1 (3.8%)
**Treatment intent** Palliative Curative	0 6 (100%)	7 (26.9%) 18 (69.2%)
**Treatment received** Surgery only Surgery + chemotherapy Surgery + radiotherapy Radiotherapy + chemotherapy Missing	0 5 (83.3%) 0 1 (16.7%) 0	5 (19.2%) 14 (53.8%) 3 (11.54%) 0 1 (3.8%)

***Primary outcome: depression and anxiety.*** Participants in the CBT group had higher mean scores on both depression and anxiety than the control group at baseline, with the difference in anxiety reaching statistical significance ( *p*=.03; see Table 2). Scores decreased to both post-intervention and follow-up in both groups, and we note they are higher in control group at post-treatment compared to those who received the intervention. As an assumption check for ANOVA tests, Levene’s tests for homogeneity of variance (taken from the 2*3 ANOVAs) demonstrated that the assumption of equal variances between groups was not violated for either depression (baseline, *p* = .157; post-treatment, *p* = .449; follow-up, *p* = .907) or anxiety (baseline, *p* = .844; post-treatment, *p* = .440; follow-up, *p* = .605). 

**Table 2.** Means and standard deviations for each variable across groups and time.

	CBT	Control	Baseline differences
**Depression** Baseline Post Follow-up	9.50 (3.99) 3.20 (2.68) 4.00 (2.83)	5.58 (5.01) 3.58 (3.20) 2.24 (3.13)	U = 40.50, p = .069
**Anxiety** Baseline Post Follow-up	8.83 (5.31) 1.80 (1.30) 2.67 (2.52)	4.08 (3.19) 2.78 (3.44) 2.24 (3.13)	U = 32.00, p = .030
**Quality of life** Baseline Post Follow-up	53.97 (4.19) 53.73 (3.54) 59.00 (1.41)	54.90 (8.04) 55.96 (8.04) 59.00 (7.01)	U = 61.50, p = .425
**Care needs total** Baseline Follow-up	9.67 (3.32) 7.50 (1.91)	10.23 (2.50) 8.00 (1.77)	U = 64.00, p = .499
**Sexuality care needs** Baseline Follow-up	7.67 (4.55) 6.25 (4.27)	4.85 (2.17) 3.82 (1.94)	U = 50.50, p = .174
**Physical daily living needs** Baseline Follow-up	10.83 (1.72) 8.50 (2.89)	11.12 (4.59) 8.59 (4.37)	U = 29.50, p = .056
**Psychological needs** Baseline Follow-up	28.43 (12.27) 20.00 (8.60)	22.62 (7.25) 18.12 (7.99)	U = 54.50, p = .256
**Health system needs** Baseline Follow-up	21.00 (5.29) 15.75 (3.95)	22.88 (7.25) 19.88 (3.44)	U = 69.50, p = .673
**Psychological inflexibility (total)** Baseline Follow-up	70.00 (29.35) 39.25 (16.38)	45.46 (12.87) 36.88 (12.85)	U = 26.00, p = .012
**CompACT BA** Baseline Follow-up	17.67 (13.60) 10.00 (5.72)	5.35 (4.53) 3.88 (4.94)	U = 24.50, p = .010
**CompACT VA** Baseline Follow-up	16.83 (7.70) 7.00 (9.20)	11.42 (6.32) 9.18 (6.78)	U = 44.00, p = .100
**CompACT OE** Baseline Follow-up	16.83 (7.70) 22.25 (8.85)	28.69 (7.99) 23.82 (9.36)	U = 60.50, p = .396
**Thought intrusion** Baseline Follow-up	7.67 (6.89) 4.50 (4.12)	4.12 (4.32) 5.18 (4.82)	U = 48.00, p = .144
**Cancer self-efficacy** Baseline Follow-up	79.50 (15.76) 93.00 (10.89)	93.12 (13.06) 96.41 (8.65)	U = 37.50, p = .050

*Note*: baseline comparisons were computed using Mann Whitney-U tests given small samples.

For depression, comparing only baseline to post-intervention changes, we found a significant main effect of time ( *F*[1, 27] = 13.43, *p* = .001, n ^2^_par _= .33), but a non-significant main effect of condition ( *F*[1,27] = .63, *p* = .435, n ^2^_par _= .02) and a non-significant time by condition interaction effect ( *F*[1, 27] = 2.44, *p* = .130, n ^2^_par _= .08). Using a 3*2 ANOVA which also incorporated follow-up scores, neither the main effect of time ( *F*[2, 34] = 1.99, *p* = .152, n ^2^_par _= .11), or condition ( *F*[1,17] = .60, *p* = .45, n ^2^_par _= .03), nor a time by condition interaction ( *F*[2, 34] = .03, *p* = .973, n ^2^_par _= .00) reached significance.

Similarly, for anxiety, comparing baseline to post-intervention scores, our data show a significant main effect of time ( *F*[1, 25] = 15.854, *p* = .001, n ^2^_par _= .39), but non-significant main effects of condition ( *F*[1, 24] = .409, *p* = .528, n ^2^_par _= .02) and time by condition interaction ( *F*[1, 25] = 5.84, *p* = .023, n ^2^_par _= .19). Extending this to include follow-up data (comparing baseline, post and follow-up) resulted in the main effect of time remaining significant ( *F*[2, 34] = 11.07, *p* < .001, n ^2^_par _= .39) and additional significant differences emerging for main effects of condition ( *F*[1,17] = .21.12, *p* < .001, n ^2^_par _= .56) and time by condition interaction ( *F*[2, 34] = 3.51, *p* = .04, n ^2^_par _= .17).

***Secondary outcomes: quality of life and unmet needs.*** Participants in the CBT group had lower mean scores on quality of life at baseline and at post-intervention than those in the control condition. Mean scores were roughly equivalent at follow-up for both groups. Levene’s tests for homogeneity of variance demonstrated that the equal variances assumption was violated for quality of life at baseline ( *p* = .034) and follow-up ( *p* = .044) (2*2 model only).

Baseline to post-intervention change in quality of life failed to reach significance for main effects of time ( *F*[1, 27] = .28, *p* = .600, n ^2^_par _= .01), condition ( *F*[1, 7] = .21, *p* = .635, n ^2^_par _= .01) or time by condition interaction ( *F*[1, 27] = .04, *p* = .847, n ^2^_par _= .00). Including follow-up data failed to improve these findings: no main effects of time ( *F*[2, 24] = .29, *p* = .749, n ^2^_par _= .02), condition ( *F*[1, 12] = .60, *p* = .453, n ^2^_par _= .05), or time by condition interaction ( *F*[2, 24] = .78, *p* = .472, n ^2^_par _= .06) effects reached significant. However, these latter results should be interpreted with caution given that there were only 2 participants in the CBT group and 12 participants in the control group.

Moderate to high needs were defined as a score of four or five on each item of the SCNS-SF34, with a score less than three indicating no need at present. Regardless of group allocation, all participants had at least one moderate-to-high need in the patient care and support needs domain. The highest rated need was ‘ *Reassurance by medical staff that the way you feel is normal*’ for the CBT group participants, and ‘ *Hospital staff attending promptly to your physical needs*’ for the control group. Given the small sample size and some violations of normality on these variables, we were unable to run parametric statistical analysis; instead, Mann-Whitney U test confirmed that there were no differences between group on overall domain scores of psychological needs, sexuality needs, health system and information needs, physical daily needs or care and support needs ( Table 2). Unmet needs reduced over time for both groups, and in all domains. A 2*2 ANOVA on total needs score demonstrated a significant main effect of time ( *F*[1, 19] = 7.9, *p* = .011, n ^2^_par _= .29), but non-significant main effects of condition ( *F*[1, 19] = .07, *p* = .799, n ^2^_par _= .00) and time by condition interaction effect ( *F*[1, 19] = .11, *p* = .109, n ^2^_par _= .01).

***Intervention mediator variables.*** Due to our limited sample size, we were unable to undertake statistical analysis of potential therapeutic process as outcome mediator variables. At baseline, thought intrusion was higher in the CBT group and cancer-specific self-management self-efficacy was higher in the control group. At baseline, the CBT group also scored higher in overall psychological flexibility and sub-scale scores of behavioural activation and value-action; interestingly, however, our control group scored as more inflexible on the openness to experience subscale. By follow-up, between group differences had minimised with the CBT group reducing in intrusive thoughts, increasing in self-management self-efficacy and reduced psychological inflexibility, though there was high variance on each, as indicated by large standard deviations.

Two-by-two mixed ANOVAs were run to explore between group differences in score changes from baseline to follow-up ( Table 3). These demonstrate no significant main effects or interaction effects for thought intrusion. A significant main effect of time was found for self-management self-efficacy, and whilst the main effect of condition failed to reach significance, the time by condition interaction was significant. For psychological inflexibility (total score), there was only a significant main effect of time only.

**Table 3.** Results from mixed 2*2 ANOVAs for potential mediator variables.

		df	df _error_	*F*	*p*	n ^2^_pa_	M (SD) Control	M (SD) CBT
Thought intrusion	Time Condition Time*Condition	1 1 1	19 19 19	.24 .03 .08	.630 .867 .359	.01 .00 .05	Baseline: 3.59 (4.18) Follow-up: 5.18 (3.82)	Baseline: 5.00 (2.83) Follow-up: 4.50 (4.12)
Self-efficacy	Time Condition Time*Condition	1 1 1	19 19 19	7.10 3.94 7.36	.015 .062 .014	.27 .17 .28	Baseline: 96.53 (8.47) Follow-up: 96.41 (8.65)	Baseline: 79.75 (20.27) Follow-up: 93.00 (10.89)
Psychological flexibility	Time Condition Time*Condition	1 1 1	19 19 19	7.10 1.72 1.33	.015 .205 .263	.27 .08 .07	Baseline: 43.12 (10.67) Follow-up: 36.88 (12.85)	Baseline: 55.00 (12.33) Follow-up: 39.25 (16.38)

## Discussion

With the potential for the benefit of psychological intervention on distress and quality of life in cancer so readily acknowledged ^13, 58^, research that expands the evidence both in terms of efficacy *and* readily implementable interventions is needed. Given the importance of cost-effectiveness ^24^, group-based, low-intensity interventions are attractive, and the growing role of Psychological Wellbeing Practitioners (PWPs) in managing the psychological effects of chronic health conditions in the UK offers an obvious route through which to test the feasibility of these types of interventions. The study, therefore, aimed to explore the effectiveness of a PWP co-facilitated, group-based, psychological intervention for improving depression and anxiety in women treated for gynaecological cancer. As secondary aims, we wanted to explore intervention effects on quality of life and unmet needs, and to identify potential psychotherapeutic mediators of intervention outcomes.

### Effectiveness of the intervention

A series of practical issues resulted in low recruitment and so our data was not sufficiently powered to address any of these aims conclusively. Nonetheless, there are some interesting patterns in the data. Baseline scores of anxiety and depression were higher in the intervention group; this is not surprising given that they had presented for additional psychological support, compared to our control group which was a population snapshot. Of importance, this probably reinforces the idea that not all cancer survivors suffer with problematic levels of psychological distress, and even in those that do, only a small minority will want this to be addressed with a formal psychology-based intervention ^59^. Depression improved over time in all participants however, no between-group differences were identified. Anxiety, in comparison, showed more intervention benefits with significant between-group differences emerging from baseline to follow-up. It is possible that the lack of statistical power is masking the true effectiveness of the intervention, but as they stand, these data suggest that the intervention might be more effective for anxiety outcomes than it is on targeting improvements in depression. Such a difference might not be surprising given the different causal factors that may be at play in the development of these distress components in cancer ^60^, and thus the different intervention strategies that might be needed.

The most frequently endorsed unmet needs in our sample were those in the ‘Patient Care and Support’ domain. This is in contrast to other studies that suggests that psychological and sexuality needs are more prominent in this survivor group ^10, 11^. Unmet needs reduced over time in both groups as might be expected as part of the natural adaptation process at the end of treatment, but we identified no additional intervention effects on this speed or extent of this improvement.

There is a paucity of research investigating potential psychological mediators in cancer survivors ^15^, therefore the EPELIT study was designed to also explore potential mediators. This is important: where the most effective mediators can be identified, intervention content can be refined to maximise change on these process variables, to consequently maximise improvements in desired outcome variables. We urge caution interpreting the results of changes in putative psychotherapeutic mediators from our study, however we are encouraged by the significant time by condition interaction for self-management self-efficacy. Given the literature elsewhere on self-management self-efficacy as a useful coping and adaptive skill ^55^, it is possible that this might have accounted for some of the improvement in anxiety in our sample. Further work to test this in larger, more powered samples, is now needed.

Although we did not collect data on intervention acceptability, this is clearly of interest given the novel facilitation method of this intervention, such as the use of a PWP intervention facilitation. Drop-out from psychological interventions can be a useful proxy indicator of acceptability and to this end, the data from this study are encouraging and suggest that CBT remains an acceptable intervention framework for this group ^15^, and that delivery by a team including PWPs was not off-putting to those who agreed to participate.

### Study evaluation and methodological discussion

Though our participant numbers were low, response rates were encouraging. Because study invitation was provided by a known member of the clinical care team, we had expected around a 70–80% participation rate ^42^, and our study data bear this out: we achieved 72 and 75% participation rates in the control and intervention group respectively. Retention to follow-up was also good with attrition of just 16% in the intervention group and a higher, but still non-problematic, 35% in the control group. Our failure to successfully recruit was not, therefore, a problem of consent or retention, but rather one of baseline population size: at our control site, the number of new diagnosis was just one third of the number that we had been led to expect on designing the trial, and at the intervention site far fewer participants were referred (or self-referred) to this intervention than had been expected, falling short of expectations by a factor of four. To further clarify, we even collected data on the reasons that patients at each site might not have been recruited. These data show that this was most definitely not an issue of high levels of ineligibility: all those presenting for the intervention group were eligible for the study and 71% of all new patients diagnosed at the control site were eligible and approached. There were some demographic and clinical biases in our sample, but these are representative of the clinical and demographic population from which we recruited. Nonetheless, efforts to recruit a more demographically diverse sample in future research would be welcomed as a way to expand the overall evidence-base in psychosocial oncology ^15^.

In this way, perhaps paradoxically, the greatest strength of our study design was also the greatest weakness. We selected a pragmatic non-randomised trial design for this study with both practical and ethical considerations in mind; these kinds of design are becoming more and more popular in clinical care research because they provide a way for existing services to be evaluated against a control group, without the ethical concerns of having to withhold a potentially beneficial intervention to which all patients *should* have access from the control group. It was, however, this design which led to the ultimate failure of the study: existing services, by their very design, have to be responsive to the populations that they serve, are subject to changes in staffing, funding and commissioning, and so iterative changes are to be expected over time. In our case, some of these changes introduced unexpected methodological challenges and potential bias.

We became aware that some of the intervention content and order was modified followed a change in staffing part-way through the study. Though this was not intended to change any of the fundamental psychotherapeutic goals of the intervention, changing content in this way may potentially have changed the macro-level focus of different underlying psychotherapeutic process. Had we recruited to target this would have undoubtedly made analysis of mediator data incredibly complex, if not entirely uninterpretable. Again, whilst understandable from a service-delivery perspective, this can have profound consequences on the methodological validity of aligned effectiveness trials.

Relatedly, with our post-intervention data point scheduled for week seven (one week after the close of the original design of the group intervention) this change to the intervention programme meant that for those participating in the study after this change, the post-intervention questionnaire assessment was completed after receiving only 75% of the intervention sessions. Our trial steering group made the decision not to change the timing of this data collection point given that a considerable number of control-group participants had already been recruited and this would have rendered their data non-comparable. Given variance in exact completion time of longer-term follow-up across the literature, we do not anticipate that this change would have affected our final three-month follow-up data, but it may well have affected earlier time-points. This highlights the challenges of evaluating existing services which have to be responsive to local clinical need, and the potential benefits of testing these kinds of research questions using an ‘additive’ model where more research control is retained over intervention content and delivery.

As a final important critical note, we would add that psychosocial research is often limited because the outcomes to which we are most interested are burdensome to measure. Unlike disease-oriented outcomes such as number of participants who experienced disease recurrence or survival, outcomes such as distress and quality of life are subjective, and thus require assessment using patient-reported measures. These measurement tools can feel burdensome and so we often attempt to minimise the length and number of administrations when designing studies; indeed, this is a common concern of ethical review committees. To investigate intervention mediators effectively, however, regular assessments are important; an important secondary research question in our study, therefore, was to establish the willingness of participants to report on these variables on a weekly basis. To this end, our study was a partial success: though we report 35% follow-up attrition, our discussions with participants indicated that a vast majority of these missing data points were about scheduling difficulties more than they were about participation fatigue. This is an important learning point from our work which demonstrates that we should not necessarily be quite so avoidant of higher-burden data collection protocols in our trial research.

### Implications and future research

Some implications from this study are somewhat obvious: because of recruitment issues, our study failed to answer conclusively whether (a) CBT-based interventions are helpful in improving psychological wellbeing and (b) whether psychological wellbeing practitioner facilitation is an effective delivery mechanism. Future research to address these important research questions is still very much needed, but a more traditional approach using a randomised controlled trial *independent of usual care* might be a more effective study design.

Of perhaps greater concern: whilst smaller than in some comparison cancer groups, increasing survivorship within gynaecological cancer means that far more women are living after treatment for this kind of cancer over time ^2^ and previous research demonstrates that unmet psychological and physical needs often remain high ^61^. Recruitment into these intervention groups should, therefore, have been simple. Our study failed to recruit, not because women attending the groups did not consent to be part of the trial, but simply because the intervention groups themselves failed to recruit. Work is needed, therefore, to understand why those with high needs are not presenting for this kind of psychological support: qualitative studies on the barriers to attendance or why this particular offering was unappealing would be helpful. Indeed, the future design of such interventions might benefit from using a co-design method of production ^62^ to ensure that what is offered is both acceptable to, and wanted by, those for who the services are created.

## Conclusions

This trial did not go to plan: although indications are that the intervention was acceptable and potentially helpful, recruitment was incredibly challenging and the iterative nature of development of the intervention rendered concrete conclusions impossible. Whilst we have demonstrated the success of some of our methodological choices (e.g. frequent data collection for designs intended to explore mediation), our overall design choice of employing a pragmatic trial design was not appropriate because of the lack of control we had over both recruitment and changes to intervention content. This has important implications for future research in psychosocial oncology; whilst pragmatic trials might be appropriate for later-stage implementation research, we found them to be fraught with methodological challenges for earlier-stage effectiveness trials and would urge others to use them with caution.

## Data availability

### Underlying data

Due to the information provided to the approving ethics committee and to individual participants at the point of informed consent, the underlying data for this study cannot be placed in an open access repository. An anonymised data set is available on request from the corresponding author. This dataset will include self-report measures, demographic information as reported to the research team, and clinical data as extracted from patient medical records; it will not include information on hospital site to minimise the likelihood of any individual participant being identifiable. Provided that the data are intended for research use (including verification of the analysis reported in this paper or for the exploration of additional research questions), requests will not be unnecessarily declined.

### Reporting guidelines

Open Science Framework: CONSORT checklist for ‘Evaluating process and effectiveness of a low-intensity CBT intervention for women with gynaecological cancer (the EPELIT Trial)’, https://doi.org/10.17605/OSF.IO/YGHNE ^37^.

Data are available under the terms of the Creative Commons Zero "No rights reserved" data waiver (CC0 1.0 Public domain dedication).
