# Supplementary material for: Evaluating process and effectiveness of a low-intensity CBT intervention for women with gynaecological cancer (the EPELIT Trial)
Source: AMRC Open Res. 2021 Mar 29;3:12. [Version 1] doi: 10.12688/amrcopenres.12971.1 (PMC11064984; doi:10.12688/amrcopenres.12971.1)
Supplement: Peer review history

# Reviewer report: Reviewer response for version 1

**DOI:** 10.21956/amrcopenres.14042.r26939

**Published:** 2022-03-11

**Authors:** Vânia Gonçalves

**Affiliations:** 1 Centre for Health Studies and Research of the University of Coimbra (CEISUC), Faculty of Economics, University of Coimbra, Coimbra, Portugal

This manuscript aims to test the effectiveness of a PWP co-facilitated intervention for reducing depression and anxiety in gynaecological cancer survivors. Secondary aims included exploring the effects of the intervention on quality of life and unmet needs.

 First of all, I would like to congratulate the authors on their effort to conduct this research despite practical issues of study implementation that they faced. Although, their study had a very small sample size and low power to reach more robust conclusions, they make a good contribution to a population of cancer survivors that has been often neglected in research. In addition, they highlighted important issues that obviously require further investigation, such as barriers that preclude patients in need to adhere to psychological support. I would recommend extending these studies to understand health professionals and services barriers that difficult the implementation of those interventions. Furthermore, authors' reflection on methodological issues of their study is important in the way that open avenues for future research targeting effectiveness of psychological interventions in these patients. As a minor point, I think authors should refer, in 'implications and future research' section, to the choice of the sample. As they mention, in their paper, there were statistical differences in age in the intervention and control groups. Obviously, this introduces biases in the analysis. Younger women face challenges that older women do not face, for example fertility and childbearing that can be very debilitating. This should be taken into account when designing future trials.

Is the work clearly and accurately presented and does it cite the current literature?

Yes

If applicable, is the statistical analysis and its interpretation appropriate?

Yes

Are all the source data underlying the results available to ensure full reproducibility?

Yes

Is the study design appropriate and is the work technically sound?

Yes

Are the conclusions drawn adequately supported by the results?

Yes

Are sufficient details of methods and analysis provided to allow replication by others?

Yes

Reviewer Expertise:

Psycho-oncology, oncofertility

I confirm that I have read this submission and believe that I have an appropriate level of expertise to confirm that it is of an acceptable scientific standard.

# Reviewer report: Reviewer response for version 1

**DOI:** 10.21956/amrcopenres.14042.r26659

**Published:** 2021-05-11

**Authors:** Peter Fisher

**Affiliations:** 1 Department of Primary Care and Mental Health, University of Liverpool, Liverpool, UK

The study reports a pragmatic non-randomised controlled study examining the effectiveness of a low intensity CBT for distress in survivors of gynecological cancers. A key outcome of the study was that sufficient number of participants were not recruited. In turn, the study was under powered and did not allow any firm conclusions to be drawn. However, as the authors note the use of the non randomised pragmatic design was a major strength as it obviates the need to withhold a potentially valuable intervention. Understanding the difficulties in recruiting to such a design will enable future studies to overcome the recruitment  issues and use a clinical valuable methodology.

 More importantly, the study highlighted that there is a need to understand why people with a level of distress that warrants a clinical intervention are not accessing available services. It is evident that a need exists to understand this issue. Overall, the study produced very valuable results which should aid the development of psycho-oncology services.

Is the work clearly and accurately presented and does it cite the current literature?

Yes

If applicable, is the statistical analysis and its interpretation appropriate?

Yes

Are all the source data underlying the results available to ensure full reproducibility?

Yes

Is the study design appropriate and is the work technically sound?

Yes

Are the conclusions drawn adequately supported by the results?

Yes

Are sufficient details of methods and analysis provided to allow replication by others?

Yes

Reviewer Expertise:

Psychooncology, mental health in physical health conditions.

I confirm that I have read this submission and believe that I have an appropriate level of expertise to confirm that it is of an acceptable scientific standard.
